# Reliability Testing of a Low-Cost, Multi-Purpose Arduino-Based Data Logger Deployed in Several Applications Such as Outdoor Air Quality, Human Activity, Motion, and Exhaust Gas Monitoring

**DOI:** 10.3390/s23177412

**Published:** 2023-08-25

**Authors:** Erik Hernández-Rodríguez, Rosa Amalia González-Rivero, Olivier Schalm, Alain Martínez, Luis Hernández, Daniellys Alejo-Sánchez, Tim Janssens, Werner Jacobs

**Affiliations:** 1 Faculty of Electrical Engineering, Universidad Central “Marta Abreu” de Las Villas, Road to Camajuaní Km 5.5, Santa Clara 54830, Cuba; ehrodriguez@uclv.cu (E.H.-R.); amguardia@uclv.edu.cu (A.M.); luishs@uclv.edu.cu (L.H.); 2 Faculty of Chemistry, Universidad Central “Marta Abreu” de Las Villas, Road to Camajuaní Km 5.5, Santa Clara 54830, Cuba; rogrivero@uclv.cu (R.A.G.-R.); daniellysas@uclv.edu.cu (D.A.-S.); 3 Antwerp Maritime Academy, Noordkasteel Oost 6, 2030 Antwerpen, Belgium; tim.janssens@hzs.be (T.J.); werner.jacobs@hzs.be (W.J.)

**Keywords:** Arduino, data logger, reliability, monitoring, sensors, custom expansion shield

## Abstract

This contribution shows the possibilities of applying a low-cost, multi-purpose data logger built around an Arduino Mega 2560 single-board computer. Most projects use this kind of hardware to develop single-purpose data loggers. In this work, a data logger with a more general hardware and software architecture was built to perform measurement campaigns in very different domains. The wide applicability of this data logger was demonstrated with short-term monitoring campaigns in relation to outdoor air quality, human activity in an office, motion of a journey on a bike, and exhaust gas monitoring of a diesel generator. In addition, an assessment process and corresponding evaluation framework are proposed to assess the credibility of low-cost scientific devices built in-house. The experiences acquired during the development of the system and the short measurement campaigns were used as inputs in the assessment process. The assessment showed that the system scores positively on most product-related targets. However, unexpected events affect the assessment over the longer term. This makes the development of low-cost scientific devices harder than expected. To assure stability and long-term performance of this type of design, continuous evaluation and regular engineering corrections are needed throughout longer testing periods.

## 1. Introduction

People use data loggers for various reasons, primarily to monitor and record data over time. Data loggers are electronic devices equipped with sensors that collect and store information from their surrounding environment. They are, among other things, used in decision support systems [1] or in early warning systems [2]. Typical parameters that can be monitored with sensors are temperature, relative humidity, pressure, the intensity of visible light, or the concentration of gaseous or particulate pollutants in the air. Motion, orientation and position of the device are also parameters often requested. A multi-purpose data logger refers to a type of data logger that is designed to be versatile and adaptable for a wide range of applications. It is a flexible device that can be configured and used to monitor and record various parameters based on the specific needs of the user. Multi-purpose data loggers are commercially available (e.g., DataTaker, Novus FieldLogger, and Campbell Scientifics), but they are rather expensive (>1000 euro). This can be a problem when data loggers are used in harsh conditions [3,4], and a short lifetime of the equipment can be expected. For some of these situations, low-cost, multi-purpose data loggers would be a better alternative. An additional advantage is that such devices are also affordable to low-income countries. To make such applications possible, the data logger should withstand a tropical climate such as in Cuba. 

Low-cost, open-source hardware such as Arduino or Raspberry Pi has found its way into the scientific community for quite some time [5]. This can be seen in the many scientific publications that make use of this technology [6,7,8,9,10,11,12,13]. For example, they are used in agriculture and livestock farming [14,15,16], to monitor the irrigation of crops [17], the respiration rate of fruits and vegetables [18], the emission of methane from animals [19,20], change of water level during tides [21], water analysis [22], urban heat islands [23], etc. Arduino-based data loggers are also used to monitor energy production (e.g., solar energy [24,25]) or consumption. It is also widely used for indoor and outdoor air quality monitoring [26,27,28,29,30,31,32,33,34]. The wide applicability of this kind of technology in research suggests that there is an interest in the development of low-cost scientific devices. 

Some of the benefits of Arduino-based data loggers are that they are cheap, customizable, and energy efficient. In addition, they can easily be replaced when they are broken. However, the mentioned advantages are also a drawback [5]. A major problem of the Arduino platform is that the user must build the system by himself. Moreover, one has to write the proper software as well. Arduino platforms do not have specific interfaces to couple sensors. For that reason, it is necessary to include a data logger shield that allows an easy connection of sensors. Different types of shields for Arduino are available on the market (e.g., Adafruit data logger shield or Grove base shield V2.0 for Arduino). Some of these shields contain a real-time clock, a memory card connector, and sometimes also different kinds of connectors to attach sensors to the data logger. However, many Arduino data loggers do not allow an easy and fast connection or replacement of sensors, as well as an easy reconfiguration of the software. For that reason, a sensor shield was designed to easily connect multiple sensors featuring analog or digital (i.e., I2C, SPI, and UART) communication outputs to the data logger using pre-existing connectors. Many people have already made software for Arduino data loggers public, but more general code that can be configured to cover a larger range of needs is harder to find. Therefore, appropriate software that includes a large set of sensors was developed, from which the user can select the sensors he needs. The versatility of the system will be illustrated with measuring campaigns related to outdoor air quality, human activity in an office, motion monitoring during a journey on a bike, and exhaust gas monitoring generated with a diesel generator. 

Besides the demonstration showing that it is technically possible to develop a multi-purpose data logger built around an Arduino Mega 2560 single-board computer, it is not a guarantee that the scientific community will use this kind of measuring device on a larger scale. The practical use of low-cost data loggers is hampered by a lack of credibility and trust. Will the data logger fail at some point, how does one know that the software is flawless, or how does one know that the sensors are generating data from which conclusions can be drawn and decisions can be made? There is sufficient literature and standards about the evaluation and calibration of sensors, but there is less information available about the evaluation of low-cost scientific devices that comprise data loggers connected to sensors. This contribution proposes an evaluation process and corresponding framework to assess the credibility of such devices.

## 2. Background

Before accepting the effectiveness of a certain technology, both rational arguments (credibility) and emotional arguments (trust) must be presented to demonstrate its proper functioning. For data loggers, this means that they should work as expected (in this case also in a tropical climate), they should protect their data, be widely used by others, and demonstrate positive experiences. The user may have perceived these positive experiences in the past, or he may have heard them from others. In addition, developers and manufacturers should be transparent about the performance and potential risks of their data loggers. This means that the believability in the proper working of data loggers is partly determined by technological features that fall in the sphere of the control of the developers but that it is also a social construct that is determined by the community using that technology. The social aspect of believing in technology falls outside the scope of this contribution. It is important to keep in mind that technology is never perfect, and there are always limitations and drawbacks, especially when using low-cost technology. Predictable errors due to known limitations are not a problem in themselves, but the boundary conditions in which low-cost data loggers can be relied on must be known in advance. In addition to the assessment process, an evaluation framework is needed to assess the credibility of a low-cost scientific device that is used within its boundary conditions. The credibility assessment process is described in Table 1. The targets that a low-cost, multi-purpose data logger must achieve (i.e., step 1 in Table 1) and the corresponding key performance indicators (i.e., step 2 in Table 1) form the evaluation framework and are classified into three groups. That framework is described in the following paragraphs. The assessment process described in Table 1 entails a continuous loop of learning, adapting, and refining, in which the reliability of a low-cost scientific device is systematically enhanced. This assessment shows similarities with the Plan–Do–Check–Act (PDCA) cycle. A drawback of this method is that there are no clear stopping criteria for cyclic assessment processes.

**Table 1:** Assessment process that was used in this work to evaluate the credibility of the multi-purpose, low-cost data logger. The corresponding evaluation framework is described in Table 2, Table 3 and Table 4.

Nr.	Assessment of the Product’s Point of View	Assessment of the Developer’s Point of View
1	Identify a set of product targets that describe a well-functioning scientific device.	Product targets act as the developer’s objectives. They describe what the developer needs to achieve.
2	Convert targets into key performance indicators (KPIs).	The KPIs act as assessment criteria for the developer’s work. They define whether the developer has realized the objectives.
3	During development, the prototype is regularly tested to see if it passes the KPIs. Failures are identified and corrected.	The developer’s work is evaluated through formative assessments. This is evaluated with small serial tests that give the developer feedback about his work process.
4	The prototype is subjected to several real-life field tests for limited time periods. Such stress tests are used to identify situations where the device fails.	The developer’s work is evaluated through summative assessments. This is evaluated with a larger test organized at the end of the work process.
5	If failures are found, then steps 3 and 4 must be repeated.	If the developer’s work does not pass all KPIs, then a new cycle in the work process is required (repeat steps 3 and 4).
6	The credibility of the device gradually increases when more evidence is found that it meets the KPIs, but it may also gradually drop when new failures are found.	The trust that the developer has in its design increases with the number of cycles it has gone through and when it takes more effort to identify a next failure.

An alternative assessment method is to identify design flaws by assessing the operation of a device for a large number of predefined scenarios describing what could go wrong, as is assessed in the failure mode and effects analysis (FMEA) [35,36]. However, this method is only applicable when all possible failures are known in advance. In such a situation, a distinction between a good and poor design can easily be made with a single test. Since the list of all possible failures was unavailable during the development stage, failures had to be discovered using the assessment method proposed in Table 1. Due to a lack of prior knowledge about possible failures and their causes, it is hard to classify a prototype as a poor design except when obvious errors are made that a well-trained engineer is not supposed to make. It should be remarked that hindsight bias (i.e., tendency of individuals to believe, after a failure has occurred, that they “knew it all along” and that the failure was predictable or obvious, even when they had no such foresight or knowledge beforehand) is not a basis to label the prototype as a poor design.

### 2.1. Targets at the Level of the Device

The Arduino-based highly customizable data logger must be designed in such a way that any kind of sensor from a predefined set of sensors can be connected to the data logger in an easy way and that the software can be configured by a user who has limited expertise in engineering without introducing errors in the system. The data logger must also withstand harsh conditions such as the tropical climate of Cuba (hot, humid, airborne salinity, and high solar irradiance) or the moderate marine climate in Belgium where condensation on colder surfaces can occur. One of the applications in this contribution is the monitoring of pollutants in exhaust gases, and this should also be considered as a harsh working condition.

In addition to the realization of the previously mentioned design goals and the absence of obvious design errors, the device targets in Table 2 must be fulfilled as well. The description of each target contains one or more KPIs. For each key performance indicator, the assessor compares the performance of the data logger against a predefined target value. The KPIs of targets 1.1, 1.3, and 1.6 are related to binary variables (i.e., pass or no pass); target 1.4 is related to a qualitative KPI, while targets 1.2 and 1.5 are related to indicators that can be quantified with a measurement. It is important to note that the evaluation of some of the indicators relies on a human decision rather than a quantitative measurement. For the quantitative variables, measurements can be compared with threshold values, but here again, the definition of the thresholds that distinguishes good from bad is often a human decision. The objectivity of the evaluation of the indicators can be enhanced with the four-eyes principle, meaning that the assessment of one assessor is controlled by a second independent person. If the data logger does not pass a test, the cause of the problem should be analyzed and corrective actions can be proposed [37]. The next cycle in the evaluation process described in Table 1 is then needed.

**Table 2:** Overview of targets and performance indicators related to the level of the device that are used by developers to evaluate their device.

Nr.	Target	Description of the Target and Key Performance Indicators to Be Achieved
1.1	System integration	Individual components, sub-systems, and software are integrated into one system without giving internal conflicts [38].
1.2	Sensor validation	Sensors meet the performance specifications as described in their specification sheets.
1.3	Bug-free software	The system contains sufficient memory to contain and run the software. The software does not produce incorrect or unexpected results, and it does not behave in unintended ways [39].
1.4	Transparency	Open-source software is written in a clear and concise way with sufficient comments and/or additional documentation (e.g., manual). Others should be able to understand the software in a fairly simple way and make improvements when needed.
1.5	Robustness in real-world conditions	The device maintains its properties throughout the monitoring campaign for environments that are of relevance to the user. The degradation rate of the hardware when exposed to real-world conditions or to accelerated life testing gives an insight into the robustness of the data logger. In this case, the data logger should handle the hostile environmental conditions such as in Cuba, which is known for its tropicalization problem (i.e., degradation rate of PCB coatings due to high T, high RH, high solar radiation, and corrosion of soldering due to high airborne salinity) [40,41,42,43]. It should handle harsh operational conditions (e.g., during the motion monitoring several wires were disconnected due to vibrations). This also entails the regular power cuts in Cuba or condensation of moisture on the hardware in Belgium.
1.6	Housing	The housing ensures that the sensors have contact with the monitored environment while providing protection against weather conditions like rain. It should meet the specifications of an IP65 casing [44,45].

### 2.2. Targets at the Level of Sensors

Once the multi-purpose data logger and its set of sensors have passed the tests of the developers as summarized in Table 2, the performance of the sensors can be assessed more in detail. There is a vast number of sensors available on the market in different price categories, but it is not always clear what sensor should be selected. For that reason, a set of sensor targets and related key performance indicators are needed to evaluate the sensor quality. Several standards that deal with the evaluation (e.g., repeatability, short-term drift, temperature sensitivity, and cross-interference) and calibration of sensors exist [37,46]. They use performance criteria as the ones summarized in Table 3 [47,48,49,50,51,52,53], although most evaluations focus on target 2.5. Although target 2.5 might be considered as the most important one, the other indicators should not be neglected. The assessments of the indicators are best performed by the user/assessor in collaboration with the developers because in some situations the system needs to be adapted to solve issues. It is recommended to retain records of original observations, derived data, and sufficient information to establish an audit trail [51].

**Table 3:** Overview of targets and key performance indicators related to the level of the sensors.

Nr.	Target	Description of the Target and Key Performance Indicators to Be Achieved
2.1	Robustness against itsenvironment	Sensors are in direct contact with the environment they are monitoring. They must withstand abnormally fast wear and tear, as well as various physical and chemical stresses such as shocks, vibrations, or a tropical climate so that their lifetime is sufficiently long to perform monitoring campaigns. Sufficiently long periods refer to typical time periods that are used during monitoring campaigns.
2.2	Response time	The sensors respond sufficiently fast to environmental changes of interest to the user with sufficiently low hysteresis.
2.3	Replicability	Sensors provide the same response under identical conditions at different time intervals for a sufficiently long period of time, or gradual changes can be mathematically corrected. When a measurement is repeated, similar results are expected, and the random error due to noise is sufficiently small.
2.4	Calibration setup	The sensors can be calibrated with a setup that generates sufficiently controllable conditions. Some calibration setups have been described in standards [52].
2.5	Similarity	The calibration method is effective when the sensor shows a high similarity with the factory-calibration, with other sensors, or with the gold standard. The similarity between sensor and reference can be expressed by the coefficient of determination, root mean squared error, mean absolute error, mean normalized bias, or the coefficient of variation [54,55,56]. The similarity determines the probability that a measurement is correct.

### 2.3. Targets at the Level of Data

Once the measuring system is available and the reliability of sensors is determined, one can evaluate the quality of the collected data. The data collected by data loggers not only contain information about the absolute value of a certain parameter but also about the dynamics of how a parameter varies over time [57,58,59,60,61]. The data targets and related key performance indicators summarized in Table 4 can assess the reliability of the collected data. It should be remarked that the data quality can pass the assessment test while it is still less than the data quality obtained by the gold standard. Data quality depends on the expectations of the user/assessor. In this contribution, the assessment follows the principles of a satisficer’s point of view, prioritizing satisfactory or acceptable outcomes rather than seeking optimal or perfect solutions (maximizer’s point of view). When the data quality is good enough to perform a task or answer a question, the system will pass for target 3.5. In principle, any kind of user can perform this assessment without the assistance of the technology developer. Based on the data quality achieved, some classify sensors in different categories [54,62]. It should be noted that a credible monitoring device must pass all the indicators mentioned in the three different levels. This contribution does not consider the quality of the organization that manages the data (e.g., traceability, clarity, and availability).

**Table 4:** Overview of targets and key performance indicators related to the level of the data and especially to the quality of the collected data.

Nr.	Target	Description of the Target and Key Performance Indicators to Be Achieved
3.1	Perceptibility	The variation of the parameter to be measured is larger than the limit of detection of the sensor and lower than its saturation point. In addition, the resolution of the sensor is sufficiently high to observe subtle changes in the environment that are of interest to the user. For example, temperature changes in oceans of 0.001 °C are highly relevant in the study of climate change. Moreover, the sensor must be sufficiently selective so that the impact of interfering environmental parameters is sufficiently small (i.e., a limited cross-sensitivity to other parameters).
3.2	Signal-to-noise ratio	The level of the desired signal is sufficiently higher than the background noise (signal > average background level plus 3 times the standard deviation) so that small changes in the trends that are of interest to the user can be observed [63].
3.3	Sensor errors	The sensor does not generate responses that have no physical meaning such as outliers, drifts, bias, or uncertainty. Such behaviors must be identified and corrected [64,65].
3.4	Completeness	The minimum data capture and time coverage, without considering the losses of data due to regular calibration and normal maintenance of the device, should be as high as possible and preferably higher than 90% [66].
3.5	Meaningfulness	Data contain relevant information needed to answer a specific question of the user or to solve a specific problem of interest to the user.
3.6	Data structure	The structure of the data file must be sufficiently simple so that a software (e.g., Microsoft Excel) or a user can read the data. In addition to a clear structure in the organization of the data, the measurements are also supposed to be at equidistant time intervals.

## 3. Materials and Methods

To illustrate the flexibility of the multi-purpose data logger, several types of monitoring campaigns were organized. The data from these campaigns will also be used to assess the credibility of the multi-purpose data logger. This section is organized according to the 3 levels of the evaluation framework described in the previous section. 

### 3.1. Design of the Low-Cost Data Logger

System integration in engineering terms refers to the process of bringing together different subsystems into one working whole. There are different methods of integration such as horizontal integration, vertical integration, star integration, etc. [67]. The method used in this research is the horizontal one, where the software connects all and communicates with all subsystems and where one subsystem can be replaced by another one with similar functionality. This way of working increases the flexibility of the overall system.

**Figure 1:**
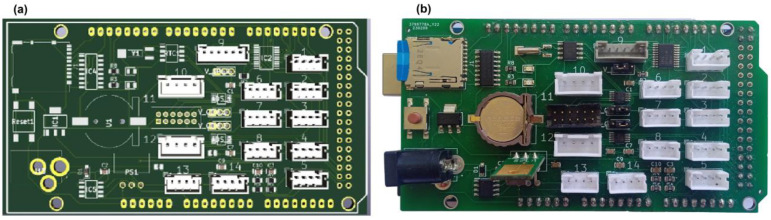
Front side of the sensor shield. (**a**) PCB design of the sensor shield as made in KiCad; (**b**) photo of the same sensor shield fully assembled and placed on top of the Arduino Mega.

Based on the previous experience of the developers [31,68,69], the Arduino Mega 2560 single-board computer was used as the core of the multi-purpose data logger. To transform the single-board computer to a data logger that can be adapted to the needs of the user, a custom-made expansion shield is needed. This shield (Figure 1) includes a real time clock (PCF8523, Manufacturer: NXP Semiconductors, City: Eindhoven, Country: The Netherlands), a micro-SD socket (10102166A812A, Manufacturer: Amphenol, City: Wallingford, Country: United States), 2 analog-to-digital converters (ADS1115, Manufacturer: Texas Instruments, City: Dallas, Country: United States) to convert the signal of analog sensors to a digital signal, a level shifter (TXS0108, Manufacturer: Texas Instruments, City: Dallas, Country: United States) to transform the output signal of the sensor in the range of 0–5 V into a signal of 0–3.3 V, and different types of JST PH and XH connectors to attach sensors to the data logger in an easy way. The connectors simplify the wiring between sensors and data logger. The electronic schematics of the sensor shield is composed of several blocks. Some of the blocks rely on the published designs from developers such as Adafruit or Sparkfun that were adapted to connect the blocks with each other. The advantage of this way of working is that such blocks have already been tested extensively by others. For example, the data storage, real-time clock, and ADC functions of the sensor shield have been taken from the Adafruit Data Logger Shield. The block related to the level shifter has been taken from SparkFun. The shield also features a 5 V 2A power supply (mezd71202a, Manufacturer: MPS Monolithic Power Systems, City: Washington, Country: United States), a 3.3 V 1A (1117 3.3) power supply, a current sensor (ACS712, Manufacturer: Allegro MicroSystems, City: Manchester, Country: United States), several jumpers for voltage selection (5 V/3.3 V) to the different connectors to power the sensors, and two LEDs for status indication. The shield provides voltage to the Arduino Mega 2560 through the Vin pin. The schematics of the shield and a photo of the front side are shown in Figure 1. The shield was designed with the open-source software KiCad and manufactured by JLCPCB (https://jlcpcb.com/, accessed on 6 February 2023).

**Figure 2:**
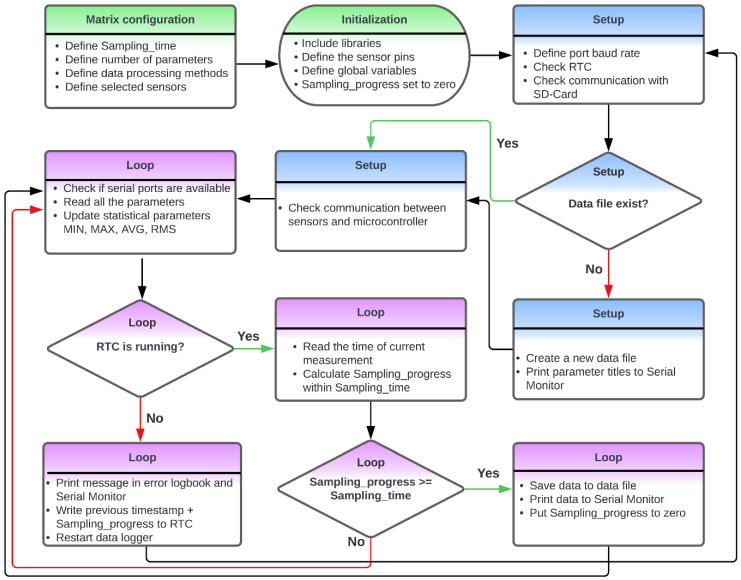
Schematic representation of the software architecture. The colors visualize the 3 mains blocks in the software: (1) initialization of variables, (2) setup, and (3) the main loop.

The software written in the Arduino IDE 1.8.19 environment is structured in such a way that the user can define the sensor type, the connector position on the shield to which it is connected, and the method with which the collected data must be processed within a sampling period (e.g., minimum value, maximum value, average, and root mean square). The user configuration is conducted in the very first step in the flowchart shown in Figure 2 (i.e., green colored boxes in the flowchart). The rest of the flowchart is a generic algorithm. The sensor-specific code is isolated from the main loop using functions. The design of the sensor shields and the software went through several evaluation cycles (see Table 1) before reaching the design goals described in Section 2.1. For each use case, the software is reconfigured to adapt it to the set of selected sensors and to the desired data processing mode. This reconfiguration results in variable memory consumption and in variable total energy consumption. To ensure that the data logger works correctly, memory-based tests were carried out. This involved checking whether the software fitted in the memory range of the device to avoid memory overload or if it slowed down the execution of the software. The software passed this target 1.3-related KPI. The results of this analysis are given in Table 5.

The power consumption of the data logger was analyzed to predict the estimated time of autonomous operation. This is an aspect of target 1.5. The information was needed to determine the appropriate battery capacity for motion monitoring. Depending on the type of measurement campaign, the data logger can be energized through a power socket of 110 VAC or 220 VAC using a power supply adapter. In the case of a mobile monitoring campaign, the data logger can also be connected to a power bank (model EY-PB-18000) or a lead battery (12VDC/7Ah). Some power banks demand a current consumption above a certain threshold, or they turn themselves off. Such power banks cannot be used with Arduino-based data loggers.

**Table 5:** Memory allocation and power consumption for each case of study.

Parameter	Outdoor Air Monitoring	Office Monitoring	Motion Monitoring	Exhaust Gas Monitoring
Used memory (bytes)	87,116	86,726	87,428	86,988
Total memory (bytes)	253,952	253,952	253,952	253,952
Global variables (bytes)	3995	3883	4307	3867
Local variables (bytes)	4197	4309	3885	4325
SRAM (bytes)	8192	8192	8192	8192
Power consumption (W)	1.60	1.15	1.62	1.25

### 3.2. Selected Sensors

Table 6 lists all the sensors that are included in the software. Different combinations of sensors were used for each of the 4 case studies. The user can define the sampling time in seconds and how the consecutive measurements within a sampling period are processed.

**Table 6:** Overview of all the sensors that are included in the software and the sensors that were used per case study.

Sensor	Parameters	Outdoor Air Monitoring	Office Monitoring	Motion Monitoring	Exhaust Gas Monitoring
ASAIR, AM2315	T, RH	x			
Adafruit, BME280	T, RH, P	x			
TERA Sensor, NextPM	PM_10_, PM2_.5_, PM_1_	x			
Alphasense, A-series gas sensors	NO_2_, O_x_, CO, SO_2_	x			
Sensirion, SCD30	CO_2_, T, RH	x	x		
E+E, EE650	Air velocity		x		
SparkFun, SEN-12642	Sound		x		
Adafruit, VEML7700	Visible light		x		
Parallax Inc., PIR sensor 555-28027	Human motion		x		
Redshift Labs, RSX-UM7	Orientation			x	
Kemet, VS-BV203-B	Vibration			x	
U-BLOX, GY-GPSV3-NEO-M8N	GPS position			x	
SST sensing, SprintIR-WF-20	CO_2_				x
SST sensing, LuminOx	O_2_				x
Atlas Scientific, EZO HUM	T, RH				x
Atlas Scientific, EZO-PRS	P				x

### 3.3. Collected Data

The software automatically generates a CSV file in which the sensor measurements are stored. The monitoring campaigns resulted in data matrices with measurements arranged in rows and parameters arranged in columns. The first column entails the timestamp. The data structure is the same for all measurement campaigns except that the columns may contain different variables, and the time between 2 consecutive measurements may be different. This maximizes the consideration of target 3.6. When an error occurs such as the inability to access a sensor, RTC, or SD card, a message with a timestamp is sent to the serial screen and, if this is possible, also stored in the error log. The data structure of the error log follows a similar structure to the data file, and the formulation of the error messages are made as actionable as possible to help the developers in tackling the problem.

**Figure 3:**
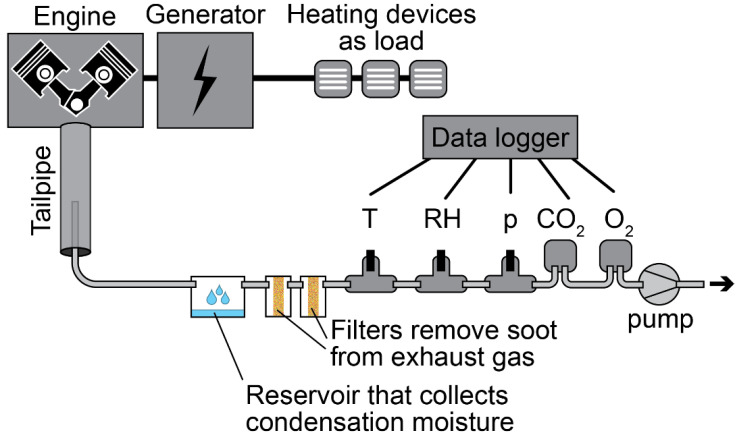
Schematic representation of the setup to monitor exhaust gases emitted by a diesel generator.

By adapting the configuration in the software, 4 different types of measuring campaigns could be realized with the same data logger. Changing the information in the configuration matrix (see the green colored boxes in Figure 2 related to ‘Matrix configuration’) is an easy procedure and only took a few minutes. The matrix consists of rows for every sensor and columns for all sensor variables (e.g., sensor type and number of the connector on the shield). The campaigns are merely illustrations to show the possibilities of a multi-purpose data logger, but they are also used for the evaluation of the credibility of the measuring device. The four different types of measuring campaigns are detailed as follows:

- **Outdoor air quality:** A measuring campaign was performed on the roof of a reference measuring station of the Flemish Environment Society (Vlaamse Milieu Maatschappij, VMM) in Antwerp, Belgium at station 42R801. The monitoring campaign was conducted from 3 to 30 June 2022. The monitoring system used a sampling time of two minutes, while the reference station used a sampling time of one hour;
- **Human activity in an office:** The activities of 3 employees working in the same office at the Antwerp Maritime Academy (AMA), Belgium were monitored from 8 to 31 March 2023. The system was placed on a table near a window. During the monitoring campaign, three workers were in the office from Monday to Friday, from approximately 8:30 to 17:30. During this period, the employees switched the lighting and electric heating device on or off. The monitoring system used a sampling time of two minutes;
- **Motion monitoring**: This monitoring campaign was performed on 17 March 2023. The parameters shown in Table 6 were recorded in the afternoon when the cyclist rode from AMA to home. In this experiment, the monitoring system was placed in a basket installed on the rudder of the bike. The monitoring system used a sampling time of one second;
- **Exhaust gas monitoring:** The exhaust from a diesel generator SD6500SS SILENT (model 186FA) with one cylinder was monitored. The generator has a nominal and peak power of, respectively, 5.7 kW and 6.5 kW. Three types of experiments were performed with a duration of fifteen minutes each and with a sampling time of two seconds. More information about the experiments can be found in Table 7. Figure 3 shows the setup used. A PTFE tube with a 4 mm inner diameter (outer diameter: 6 mm) was placed inside the tailpipe of the generator. This pipe was connected to a reservoir that collected the condensation moisture. A HEPA filter inside a Swagelok stainless steel tee-type particulate filter (SS-6TF-MM-05) was used to remove the soot before the exhaust gas reached the sensors. Screwable sensors in a stainless-steel Swagelok tube fitting with female branch (diameter sensor of 1/4 inch: Swagelok SS-8F-K4-2; diameter sensor of 3/4 inch: SS-12-T) and flow through sensors for CO_2_ and O_2_ were used to connect the sensors with the tube. The sensor types used in this campaign can be found in Table 6. A pump sucked the exhaust gases through the tube, condensation reservoir, and the filters.

**Table 7:** Overview of all exhaust gas monitoring experiments and changes in operational conditions. The letters correspond to the ones mentioned in Figure 11.

Parameters	Experiment a	Experiment b	Experiment c
Duration [minutes]	15	15	15
Fuel type	Normal diesel with less than 10 ppm of sulfur	Normal diesel with less than 10 ppm of sulfur	Normal diesel with less than 10 ppm of sulfur
Pump in gas extraction setup	No	Yes	Yes
Load on the generator	No	No	Yes
Change RPM of the generator	No	No	Yes

## 4. Results

### 4.1. Outdoor Air Quality

The data logger and the selected sensors appeared to work well in the moderate marine climate of Belgium during an outdoor air monitoring campaign on the roof of a reference station of VMM. Also, for a measuring campaign in a tropical climate such as in Cuba, the system appeared to perform well [70]. During short-term tests, the system passes targets 1.1 to 1.6. However, short-term experiments in real-world conditions resulted in insufficient information to evaluate target 1.5. During longer experiments, the data logger seemed to malfunction at some moments during the Belgian autumn and winter period. On one occasion, the connection with the RTC was lost, and no timestamps were recorded. At another moment, data were sent to the serial screen but were not stored on the SD card. When the device was taken to the office, both problems appeared to be solved by itself. Most likely, the formation of condensation during sudden meteorological changes was the cause of the problem. This problem could not be solved by better housing because the sensors must remain in contact with the open air while the box must protect the hardware against rain and other weather conditions. An alternative would be to place all hardware in a completely sealed box where the sensors inside the box are connected with each other and to the outdoor air using a PTFE tube through which air is pumped, but this is a drastic change in the design of the monitoring system. Instead, the problem was tackled by spraying varnish on the surface of the single-board computer and the sensor shield, but additional long-term tests during the autumn and winter period of next year are needed to evaluate this solution. This means that the evaluation of some targets is affected by the occurrence of several unforeseen scenarios. Such situations are known in cognitive psychology as black swan events (i.e., a metaphor that describes an event that comes as a surprise because it is beyond the developer’s imagination, has a major effect, and one tends to think it was predictable once it has occurred) [71]. For these events to take place, testing should be carried out over extended periods of time under different kinds of situations, but even then, it is not certain that such events would occur during the test phase. Unexpected events have a larger impact on the reliability that users attribute to the data logger rather than predictable errors resulting from the shortcomings of low-cost technology. The occurrence of hard-to-predict events or events that are not supposed to happen makes it not possible to assess the reliability of the data logger during the designing stage of a device. A temporal absence of unexpected events might lead to overconfidence by the developers. 

The variations of most of the environmental parameters as registered during the measuring campaigns fitted within the range that the sensor can measure. When the system was working properly, no missing data in the time series were observed. Moreover, the sensors appear to be withstanding the outdoor conditions to which they were exposed and seemed to be sufficiently robust (target 2.1). However, the TERA sensor for particulate material did not operate correctly during the measurement campaign due to a fracture in the housing of the sensor. The response times corresponded to those provided by the manufacturers (target 2.2). The BME280, AM2315, and Alphasense sensors were calibrated using a high-end calibration method at VITO, Belgium [70]. The Sensirion SCD30 CO_2_ sensor was calibrated using a low-cost method [32]. For all sensors, laboratory calibration tests have shown a strong linear relationship between sensor signal and corresponding physical values. On the basis of the short-term calibration tests, the sensors pass the targets 2.3 to 2.5. However, long-term calibration tests are needed to get an insight into their reliability over a longer time scale.

**Figure 4:**
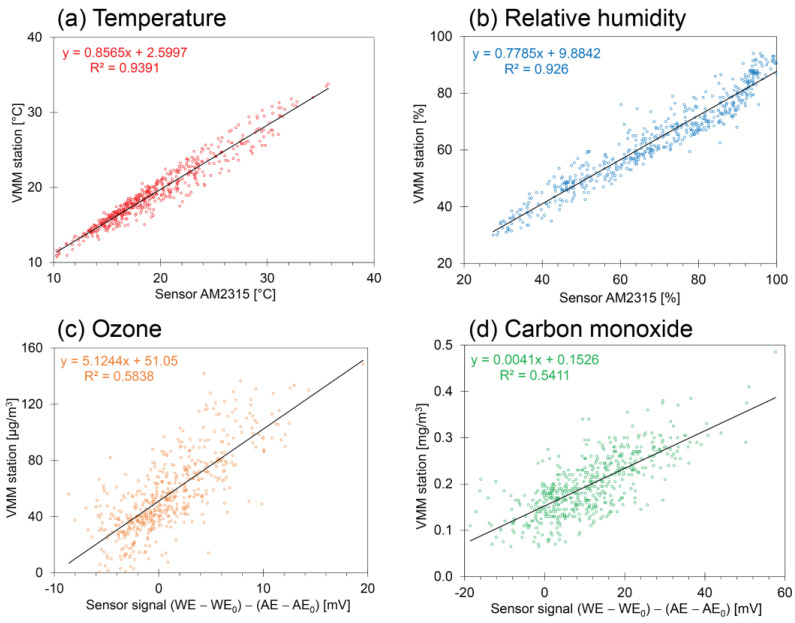
Relationship between sensor data and reference data for several environmental parameters collected in outdoor air in Antwerp, Belgium at reference station 42R801. The coefficient of determination gives an insight into the similarity of target 2.5. The symbols WE and AE signify the signals of the working and auxiliary electrodes of the gas sensor, and the subscript zero refers to zero air without any pollutants.

Most researchers use field tests to confirm the usefulness and performance of low-cost scientific devices [72]. Some of them compare such devices with state-of-the-art devices by operating them side by side in the field [73]. Such campaigns can be considered as long-term calibration tests. For air quality, co-location experiments can be performed nearby reference stations [74,75,76]. The results of such an evaluation are seen in Figure 4. In this study, the relationship between the sensor signal (i.e., a quantity generated using a digital sensor, or a voltage generated using an analog sensor) and the corresponding quantity provided by the VMM reference station was explored (see Figure 4). High coefficients of determination were obtained for temperature and relative humidity reported with the AM2315 sensor (0.9391 and 0.926, respectively). For O_3_ and CO, the coefficients of determination are lower (0.5838 and 0.5411, respectively). A similar relationship was studied in a recent investigation for SO_2,_ but for that pollutant, the concentrations at the measuring location were below or close to the detection limit of the sensor [70]. Due to the lower coefficients of determination, the simple linear calibration introduces an uncertainty in the calculated concentrations. However, the credibility of the monitoring system is not only determined by the similarity between sensor data and reference data as shown in Figure 4 but by the entire system as suggested in the previous paragraphs.

**Figure 5:**
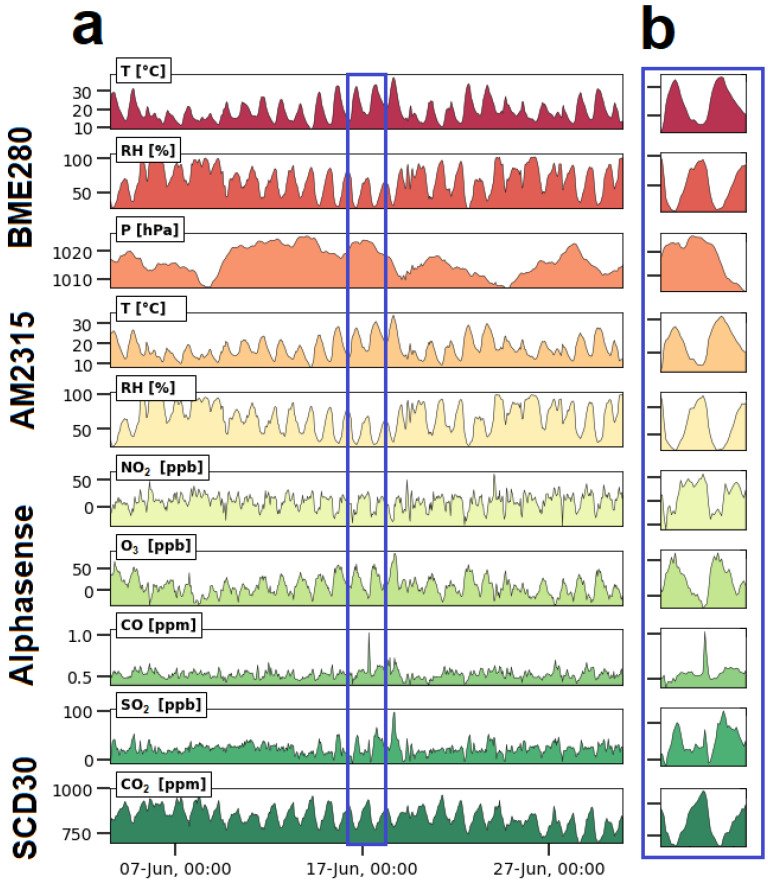
Time series plots of the outdoor air monitoring at the VMM station in Antwerp, Belgium using a low-cost monitoring system from 3 to 30 June 2022. The plots show the occurrence of meaningful trends in the graphs, from which information can be extracted. (**a**) Plot of the entire measurement campaign; (**b**) Detail of the measurement campaign located in the blue rectangle in figure a.

The trends in Figure 5a show the overview of the outdoor air measuring campaign of about 1 month in Antwerp, Belgium. The detail in Figure 5b shows the occurrence of two temperature peaks over a period of two consecutive working days and a valley during the night in between these days. Figure 5b also shows a sharp peak in the CO concentration around 17 June at 00:00. This behavior is also observed for SO_2_. Since this peak is not observed in the reference data, it is likely that it is caused by a sensor error or by cross-interference, but it might also be possible that the peak was observed due to the smaller sampling time (two minutes for the Arduino device vs. one hour for the reference data). The RH shows peaks that correspond with the temperature valleys. This illustrates that the trends in Figure 5 are day/night cycles. The recorded ozone peaks are closely related to the temperature peaks, a behavior that was also observed in other studies [77,78]. NO_2_ and CO_2_ peaks match with the relative humidity peaks and temperature valleys. In addition, some SO_2_ peaks match with CO peaks. The deviations from the linear relationships shown in Figure 4 suggest that the absolute amount of a quantity is associated with a relative high uncertainty, but Figure 5 suggests that meaningful information can be extracted from the trends.

### 4.2. Human Activity in an Office

**Figure 6:**
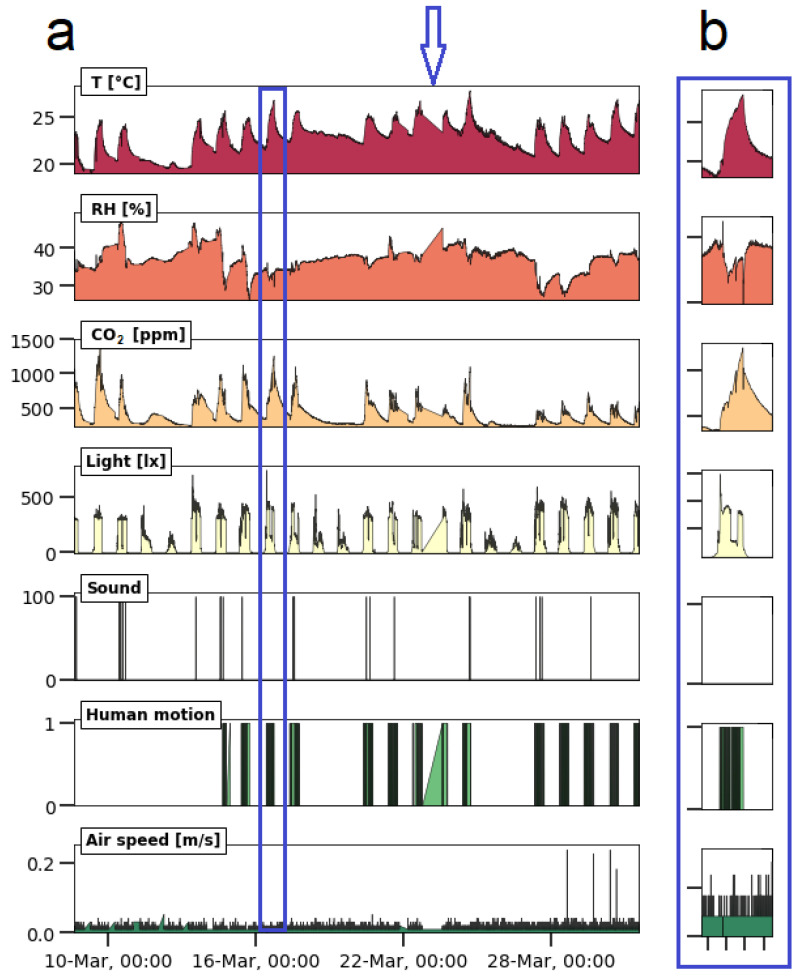
Results of the office monitoring at the Antwerp Maritime Academy using a low-cost monitoring system from 8 to 31 March 2023. (**a**) Overview of the campaign; (**b**) detail of a single workday.

The same data logger and the same software were used to set up a monitoring campaign inside an office. The sensors were selected to monitor human presence and human activity in the office and to monitor the effect of the ambient conditions on wellbeing. This means that besides temperature and relative humidity, also CO_2_, visible light (from sunlight and lighting), motion (when a person moves in the room), sound and air speed (draft inside the office) were monitored. The data logger and sensors selected for this study appeared to work without conflicts. This suggests that the system passes targets 1.1 and 1.2. However, during the first days of the campaign, a bug was detected in the software, as no data were recorded from midnight to 6:00 a.m. This moment occurred at the blue arrow in Figure 6. The problem was solved by correcting the algorithm. Another issue was a gap in the data between Wednesday, 22 at 16:00 and Thursday, 23 at 13:40, which was preceded by a measurement with an erroneous timestamp “165/165/2165”. This error can be caused by a power loss of the RTC battery or synchronization troubles between the RTC module and the Arduino. To solve this issue, an additional code was introduced in the loop that (a) checks if the RTC is working properly; (b) estimates the current time from the previous time measurement and the time that already passed within the current sampling period, writing that time to the RTC; and (c) restarts the Arduino to clean all the registers. In addition, the variables in which the RTC readings are stored were changed from integer to long. Since then, the device has been working without intermissions (target 1.3). This problem showed again that assessing the reliability of a low-cost data logger requires real-world tests of longer time spans and that unforeseen scenarios affect the assessment. However, there are no criteria that clearly delineate the concept of “long periods” in target 2.1. The data logger was not enclosed in a housing for this experiment, and target 1.6 could not be evaluated.

The sensors for visible light, sound, air speed, and human motion detect human activity in different ways. For these sensors, the targets of Table 3 were harder to evaluate because there was no access to reference equipment to calibrate this kind of sensor (i.e., target 2.4). All sensors involved in this application (see Table 6) were calibrated by the manufacturer, and/or calibration information is provided by the manufacturer. However, by changing the conditions in the room in a controlled way (e.g., switching on the light or switching on the heater), it was possible to observe the behavior of the sensors. The air speed values provided with the EE650 sensor that gives an insight into air draught were below the detection limit (i.e., 0.1 m/s), which means that the collected data were not reliable (i.e., target 3.1). All other sensors recorded values above their detection limits. The sensors did not endure signal saturation (i.e., target 3.1). The SparkFun Sound Detector indicated only the occurrence of sound above a certain threshold value, showing values of 0 or 100, where “0” means no sound detected and “100” means sound detected. The default configuration for this module is 40 dB in gain terms. The binary response was not ideal for analyzing trends of sound intensity. The motion sensor was included in the monitoring system on March 14 and was able to detect moving objects in the room, showing values of 0 or 1, where “0” means no movement detected and “1” means movement detected. Also here, a sliding scale might have given more information. 

The trends in Figure 6a show the dynamics of the measured parameters for several days. Figure 6b shows a detail of a single workday and how some of the monitored parameters are related. The temperature fluctuated so strongly because the heating device was switched on at the beginning of the workday and switched off at the end of the day. During the weekend, the heating device was not used. Relative humidity was more stable, but increases were noticed on rainy days. Wet clothing introduced moisture in the room. The CO_2_ concentration raised when people were present in the room. During the absence of people on weekends, no CO_2_ peaks were observed. The reported CO_2_ concentrations can be used as an indicator to evaluate the indoor air quality (IAQ), air exchange rate, and the ventilation efficiency in the office [79,80]. Also, visible light, sound, and motion gave an insight into human activity in the room. In what follows, detailed information is given for the analyzed parameters. There is a clear relationship between human presence/activity and the change in the parameters of temperature, CO_2_, lighting, and motion. Sound contained less information about human presence than expected, and the air speed sensor was not able to register draught (no windows were opened during the cold season). The trends provided by the sensors allowed relevant information to be drawn from the raw data, so that the system passes for targets 3.1 to 3.5.

- **T, RH, and CO_2_:** While the relative humidity was more or less stable, the temperature was the lowest in the morning and increased at around 9:00 when the workers switched on the heating device. The temperature slowly increased during the course of the day and started to decrease at around 17:30 when the heating system was turned off. The CO_2_ concentration showed a similar behavior, and its variation may be related to the number of people frequenting the office on that day. When three people are working in the office, the CO_2_ concentration peaks at around 1000–1200 ppm; with two people in the room, the peaks reached 800–1000 ppm. If the concentration of CO_2_ exceeds the 900 ppm threshold, an efficient ventilation system and the presence of air purification devices are recommended. This was especially the case during the COVID-19 period. In Figure 6b, a T peak is clearly visible. Switching on the heating device went along with a sudden drop in RH and reached higher values again when it was switched off. This anti-correlation is the normal behavior of a closed room where temperature fluctuates and the absolute amount of moisture is constant;
- **Light:** Light intensity increases as dawn rises. The light sensor clearly sees the sunlight that enters the room through the window, resulting in diurnal cycles (i.e., the large bumps). For a short period of time, the sunlight was also able to shine directly in the room, resulting in a high peak in the morning. In addition, the sensors also registered elevated intensities when the lighting was switched on in the morning, showing a sudden increase at around 8:30. After that, it remained constant. At around 12:30, the light intensity suddenly decreased because the office light was turned off during lunch time. Sometime later, the light was turned on again. A decrease in light intensity was also recorded around 17:30 p.m., when the workday ended. The intensity decreased as evening approached. This means that the light sensor gave additional information about human presence in the office;
- **Sound:** The sound peaks were recorded during working hours at random moments. This means that either the sensor was not sensitive enough to pick up the low sound levels, or that the employees in the room were working in silence. The sound peaks were related to human presence, but in this working context, it did not give much meaningful information about human context. For example, Figure 6b is a working day with human activity (i.e., see temperature, CO_2_, visible light, and human motion), but no sound peaks were registered that day;
- **Motion:** The sensor recorded human motion during working hours and was related to the presence of people in the office. No motion was recorded at night, after working hours, on weekends, and during lunch time (see Figure 6b). The valley for light during lunch time in Figure 6b is larger than for motion, which may be attributed to the fact that the office lighting was turned on later. During the periods of human presence, the sensor suggested the absence of any motion, meaning that at regular occasions, the employees were sitting still or not moving within the detection angle and distance.

### 4.3. Motion Monitoring during a Journey with a Bike

**Figure 7:**
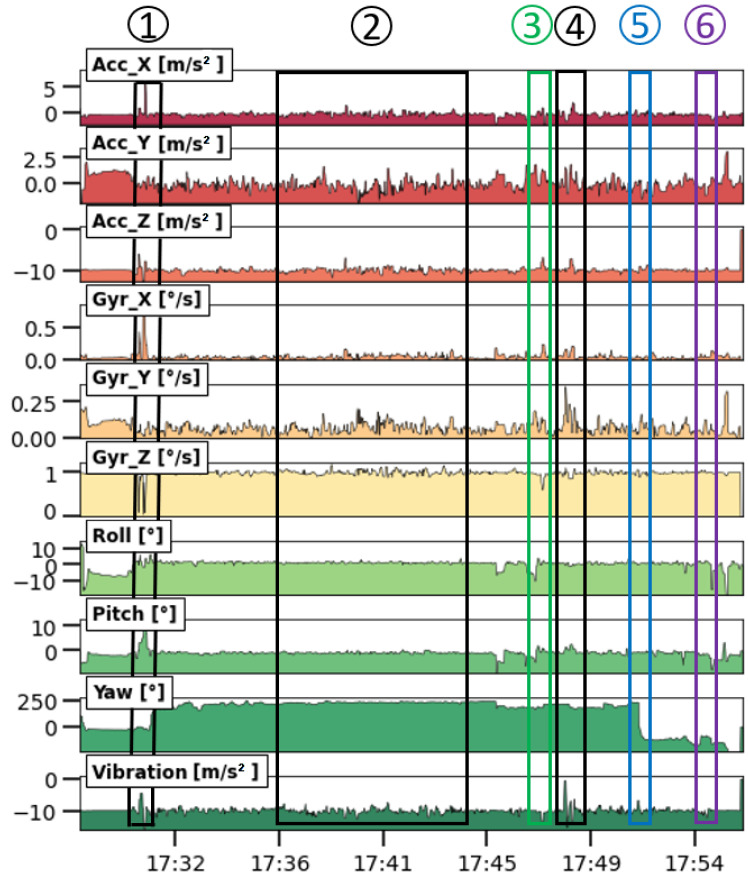
Results of motion monitoring during a bike journey from AMA (i.e., the start point of the time series) to home (i.e., end point of the time series) using a low-cost monitoring system on 17 March 2023. The marked and numbered rectangles in Figure 7 refer to the 6 documented events in the trajectory as shown in the map of Figure 8.

The same data logger was connected to another set of sensors to monitor the position and orientation of a journey on a bike. The data logger and sensors selected for this campaign worked without conflict. The software appeared to be error-free. This campaign did not operate for a long period (about 30 min), but the device worked as expected in dry outdoor conditions. In order to have optimal GPS readings, no specific housing was used in the first journey (Figure 7), but it also worked properly in a closed plastic housing during the second journey (not shown). The device appears to pass all targets from 1.1 to 1.6. 

During the journey, the integrity of the monitoring system was not damaged by vibrations (i.e., quasi periodical motion of the bicycle in the vertical direction when riding on, for example, a cobblestone road) and shocks (i.e., one-time sudden motion in the vertical direction) caused by the movements of the bike (target 1.5). A sampling time of 1 s was used to register the variations during the campaign. The vibration and orientation sensors registered several changes in the measured parameters when the cyclist encountered cobblestone roads, potholes, etc. The quality of the sensors was evaluated with the data from the measurement campaigns, where documented events that occurred during the journey should cause changes in the measured signals. However, targets 2.3 to 2.5 remained hard to evaluate from the collected data. Concerning the calibration of the sensors, this was already set up by the manufacturer, and/or the manufacturer provided calibration information.

**Figure 8:**
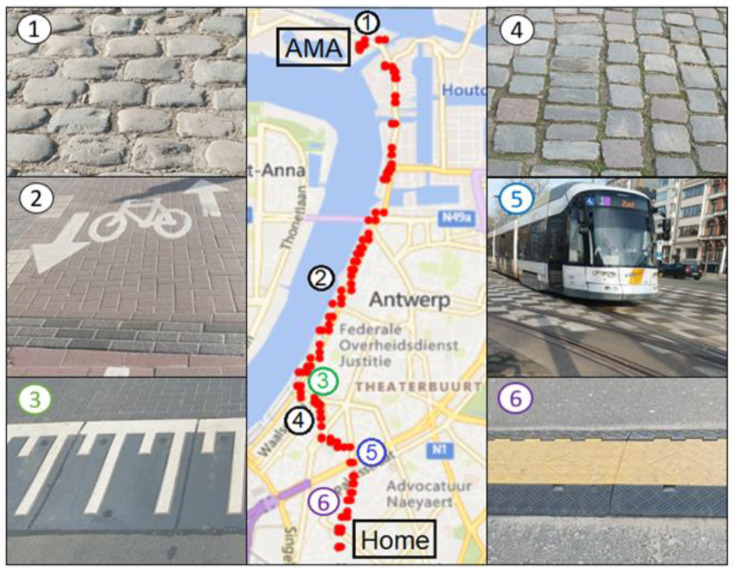
Journey of ca. 5.5 km from AMA to home using a bike at an average speed of 11 km/h together with the position of the events caused by irregularities in the road. The red dots stand for cyclist’s position according to the GPS sensor GY-GPSV3-NEO-M8N. The numbered images at the left and right are associated to the numbered positions in the map and are related to the numbered events in Figure 7.

In Figure 7, the results of the journey are visualized as a time series. The same results are also shown in Figure 8 on a map. The GPS data couples both representations of the same data. At first glance, the signals show strong fluctuations, and it is not clear whether the high frequency variations contain meaningful information or that they should be considered as noise. Since the sampling time is small, the minimum, maximum, average, and root mean square was almost identical to the raw data. Therefore, it was decided to evaluate the sensors using the raw data only. The time series contained, at regular occasions, spikes of 1 data point with unrealistic values. It was decided to remove such spikes by applying a centered-moving median with a window size of 5 points. This means that the system did not pass entirely target 3.3 but that the problem could be solved during post processing. Finally, no data gaps were observed during the measuring campaigns for vibration and orientation sensors, except for some positions without GPS measurements (see zones without red dots in Figure 8). It was not clear if this behavior was related to the response time of the sensor in relation to the fast sampling time or to the surroundings that blocked the GPS satellite signal.

The orientation sensor entails a three-axis accelerometer, gyroscope, and compass and is able to describe the orientation or rotation of an object in a three-dimensional space through the angles roll, pitch, and yaw [81,82]. The gyroscope measures the angular velocity or rotation rate of an object around an axis without a fixed reference point. The magnetometer measures the strength and direction of the terrestrial magnetic field and gives an insight into the absolute orientation. All these parameters are included in the multi-purpose data logger and can be used to monitor the motion of, for example, submarines [83], ships, or airplanes. To understand the meaningfulness of the orientation and vibration sensors, the relationship between the events (see numbered rectangles in Figure 7) and the obvious irregularities in the road that were encountered during the journey (see Figure 8) was studied. The GPS coordinates at the irregularities shown in Figure 8 were used to label the moments in the time series. These moments corresponded with peaks and valleys in the time series, thus resulting in observable signal changes. 

**Figure 9:**
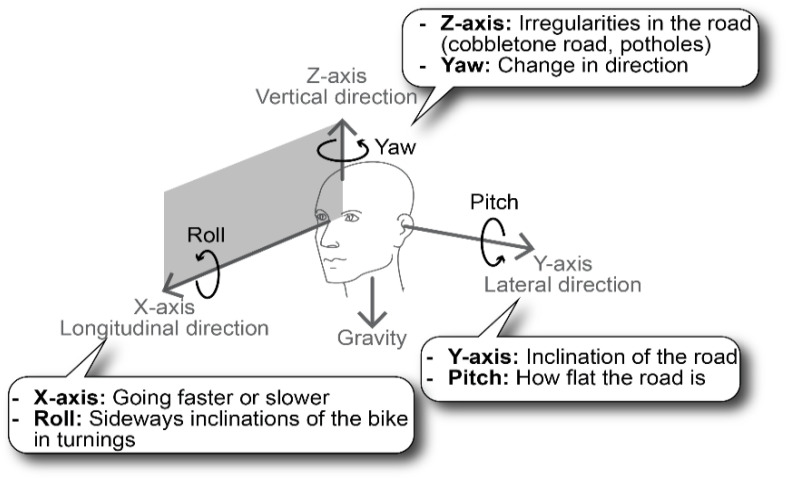
Schematic overview of the six degrees of freedom—forward/back, left/right, and up/down—including the rotations along each axis roll, pitch, and yaw (Euler angles).

**Figure 10:**
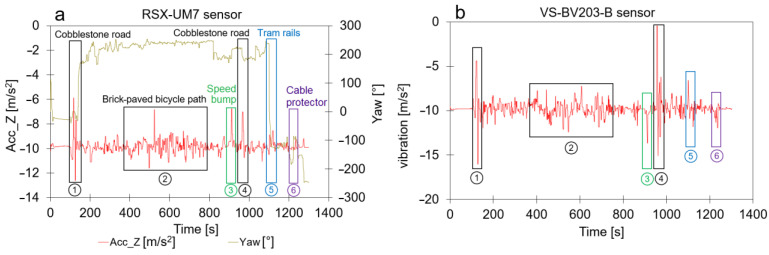
The monitoring of a journey on a bike shown as a time series collected by two different sensors. The numbered events shown in Figure 8 are also indicated in this plot; (**a**) Time series obtained by the accelerometer in the Z-axis using RSX-UM7 (it is also able to monitor the yaw); (**b**) Time series as measured by the vibration sensor VS-BV203-B.

Whether the collected data contain useful information about the motion and orientation of the cyclist, a more detailed analysis of the sensor measurements was made. For the sake of simplicity, gyroscope information is shown in Figure 7 but not discussed because the orientation obtained from the measurements (expressed in Euler angles, see Figure 9) is of greater importance. The list below describes the behavior of the sensor measurements installed on the bicycle. The trends in Figure 7 contain meaningful information about the motion of the bicycle. This means that motion monitoring can provide contextual data that can be useful for, for example, indoor air quality monitoring campaigns on board ships where pollution peaks might be explained by sudden changes in motion [84]. The sensor measurements are detailed as follows:

- **Longitudinal or forward direction:** During the journey, the Acc_X plot in Figure 7 (i.e., the acceleration in the forward direction as shown in Figure 9) is most of the time close to zero because the cyclist moved at a more or less constant speed, or he was standing still (e.g., at a traffic light). Valleys are observed when he slows down; peaks are caused by an increase in speed. During a traffic light within the period of event 4, a negative peak (brake) is followed by a a positive peak (pull). Trends in roll (i.e., rotation along the forward direction) varied around 0° because the bike was oriented vertically while riding. At some moments, negative peaks in roll are observed, which indicated an inclination of the bicycle to the left with respect to the vertical plane (Figure 9). Conversely, positive peaks indicated the moments when the cyclist was inclined to the right. GPS coordinates confirm that such peaks correspond with moments that turns were taken or that the cyclist was standing still and stood on his left foot. The six events in Figure 8 did not have a major impact on the forward direction;
- **Lateral or sideways direction:** The accelerometer values along the Y-axis (Acc_Y in Figure 7; see Figure 9 for the orientation) show the occurrence of small peaks and valleys around a zero baseline. When a bicycle leans into a turn, the cyclist endures a lateral force, and this results in spikes in Acc_Y. The scale of the vertical axis of Acc_Y is smaller than that of Acc_X in Figure 7, and this gives the illusion that there are more irregularities in the sideways direction. These variations are related to level changes on the road that the cyclist travels and to turns. During the experiment, the pitch values fluctuated around a constant value near 0° because the journey was mostly flat. However, this constant value deviates from zero and is slightly negative because the orientation of the sensor on the bike was not perfectly flat. The negative and positive peaks are related to facing a downward slope at the departure of the journey or bumps and potholes in the road. The standard deviation of the pitch reflects the different kinds of surface roughness of the road (Figure 8). The irregularities of events 1, 3, 4, 5, and 6 resulted in peaks in the pitch;
- **Vertical direction:** The vertical direction in the Z-axis was monitored with the accelerometer RSX-UM7 and with the vibration sensor VS-BV203-B. Figure 10 shows the behavior of both sensors and the response to the six events. Both sensors recorded a fluctuating signal with high standard deviation when the cyclist was riding on cobblestone roads (events 1 and 4 in Figure 8). Also, an increase in the standard deviation of the acceleration was observed when the cyclist was riding on a brick-paved bike path (event 2). Events of a shorter duration were observed in the acceleration measurements when the cyclist crossed a speed bump (event 3), tram rails (event 5), or a cable protector on the road (event 6). A comparison between both sensors suggests that the vibration sensor VS-BV203-B is more sensitive, resulting in a more pronounced signal for the six events (Figure 10). The orientation sensor RSX-UM7 improved the identification of the events by combining several parameters such as the yaw (Figure 10a). In addition, the RSX-UM7 is capable of providing all the information required for the implementation of an inertial navigation system (INS). This additional information of position and velocity can compensate for small gaps in the GPS signal. The yaw (i.e., angle measured relative to the earth’s magnetic north) changes each time the cyclist turns and maintains the direction in which he turned. Positive and negative angles refer to the clockwise and anticlockwise rotation relative to the reference plane. A large change from about +200° to −180° is actually a change in direction of about 20°. This is seen as a step in Figure 10a. When a cyclist rides around a pothole and returns to his original course, a spike is produced (Figure 10a). This behavior could be observed within events 1, 3, and 5 in Figure 10a. Changes in direction as observed in the yaw are accompanied by a change in roll. This is a common occurrence in the movement of a bicycle, where the cyclist leans slightly towards the side when he intends to turn.

### 4.4. Exhaust Gas Monitoring

For this experiment, the sensors coupled to the data logger were connected to an exhaust gas extraction setup (see Figure 3). To avoid problems with the sensors, the extraction setup removed soot and condensation when the gas was cooling down. The blackening of the second filter gave an indication when soot penetrated through the first filter. Linking the two systems worked well for periods of less than 15 min. For this application, the monitoring setup passed targets 1.1 up to 1.5 for short-term experiments. However, one might ask how to remove condensation and soot for longer periods while the system autonomously monitors exhaust gases. Housing was not needed at this early design stage (target 1.6) and for that reason could not be evaluated. 

**Figure 11:**
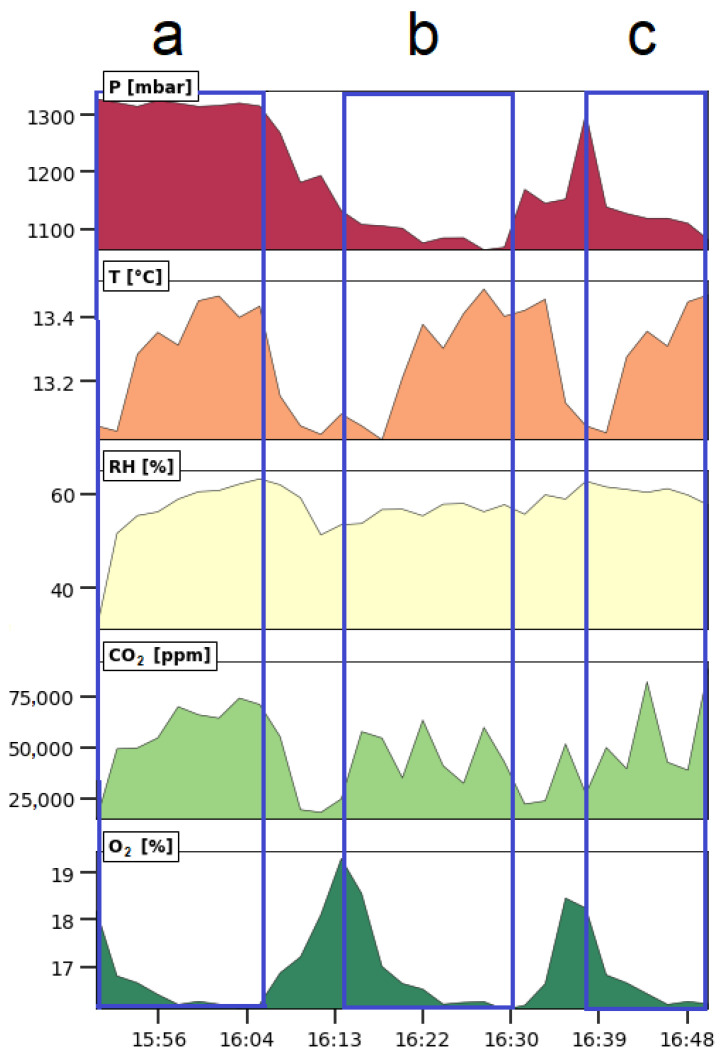
Results of exhaust gas monitoring using a low-cost gas conditioning system and a low-cost monitoring system. The 3 consecutive experiments were performed on 5 March 2023. For each experiment, a clean filter was used. (**a**) Experiment using normal diesel but where the pump in the exhaust gas extraction setup was not switched on; (**b**) same experiment as previous but where the pump was switched on; (**c**) experiment at different loads.

The short experiments did not have sufficient information to assess target 2.1. By changing the conditions of the experiments in a controlled way, it was possible to evaluate the response time of the sensors. The sensors responded sufficiently fast to detect all changes (see Figure 11). Therefore, the data logger passed target 2.2. Targets 2.3 to 2.5 could not be evaluated because the P, T, RH, CO_2_, and O_2_ sensors were not calibrated by us. However, all sensors were calibrated by the manufacturer, and/or the calibration information was provided by the manufacturers. The values recorded in this application were above the detection limit of the sensors. The SprintIR-WF-20 CO_2_ sensor measures CO_2_ concentration up to 20%, and this appeared to be too low at certain moments (target 3.1). There were no missing data in the time series (target 3.4). Non-dispersive infrared (NDIR)-based sensors analyze gasses by measuring the absorption of a light beam with a specific wavelength. The law of Lambert–Beer gives the relationship between the extent of absorbed light and the number of gas molecules in the light beam. However, the number of CO_2_ molecules in 1 m^3^ of air also depends on the atmospheric pressure [85]. The exhaust gas pressure inside the tube is affected by the pump in the gas extraction setup, and this must be considered when calculating the concentration of the gas. The formulas needed to calculate the CO_2_ concentration are mentioned in the data sheet of the sensor [86]. Therefore, the concentration recorded with the sensor must be corrected using Equation (1). Depending on the CO_2_ concentration level, the compensation factor *Y* is calculated using either Equations (2) or (3). The factor *Y* is calculated using Equation (2) when the recorded concentration *c_r_* is smaller than or equal to 1500 ppm. If *c_r_* > 1500 ppm, Equation (3) is used to calculate *Y*.

(1) $$c_{a}=\frac{c_{r}}{1+Y\left(1013-P\right)}\;\;\;\;\;\;$$

where *c_a_* is the adjusted CO_2_ concentration expressed in ppm; *c_r_* is the concentration recorded using the sensor expressed in ppm; *Y* is the compensation factor; and *P* is the pressure in the gas extraction tube expressed in mbar and measured with the Atlas Scientific sensor.

(2) $$Y=a_{0}+a_{1}c_{r}{}^{}+a_{2}c_{r}{}^{2}+a_{3}c_{r}{}^{3}+a_{4}c_{r}{}^{4}\;\;$$

$$\begin{array}{lll} a_{0}=-9.8754\times 10^{-4} & a_{2}=1.7391\times 10^{-9} & a_{4}=2.6661\times 10^{-16}\\ a_{1}=-1.2556\times 10^{-6} & a_{3}=-1.1146\times 10^{-12} & \end{array}$$

(3) $$Y=a_{0}+a_{1}c_{r}{}^{}+a_{2}c_{r}{}^{2}+a_{3}c_{r}{}^{3}+a_{4}c_{r}{}^{4}+a_{5}c_{r}{}^{5}+a_{6}c_{r}{}^{6}\;\;\;\;\;\;\;\;\;$$

$$\begin{array}{llll} a_{0}=-1.471\times 10^{-3} & a_{2}=2.311\times 10^{-14} & a_{4}=1.304\times 10^{-25} & a_{6}=2.811\times 10^{-38}\\ a_{1}=-2.195\times 10^{-9} & a_{3}=-8.126\times 10^{-20} & a_{5}=-9.817\times 10^{-32} & \end{array}$$

The trend in Figure 11 gives an insight into how the chemical composition of the exhaust gas emitted by the diesel generator varied over time during the three consecutive experiments. This means that a low-cost monitoring system can be used to collect meaningful information about exhaust gas emission:

- **Experiment (a):** At the beginning of the first experiment (see the blue rectangle on the left side in Figure 11), the generator was off, and the sensors showed values that are characteristic for the ambient conditions of the site: approximately 1300 mbar pressure, 13 °C temperature, 25% relative humidity, 7000 ppm CO_2_, and 19% O_2_. The ambient CO_2_ concentration was clearly too much for ambient conditions, but the sensor was optimized to measure high concentrations, so the measurement of lower concentrations was less accurate. It is also possible that the CO_2_ that was generated using the generator during preliminary tests penetrated the room by diffusion. A few minutes later, the generator started. Since the pump of the extraction setup remained off during the entire course of the experiment, the pressure remained constant. The exhaust gas diffused across the tube and reached the sensors. The relative humidity and CO_2_ concentration increased up to 62% and 75,000 ppm, respectively, while O_2_ dropped by 2%. These observations fit with the burning process of fuel where CO_2_ and H_2_O are produced, O_2_ is consumed, and heat is released. In this experiment, three CO_2_ peaks were observed because the generator was turned on and off three times. Since the exhaust gas inside the tube was replenished through a slow diffusion process, the width of the peaks is larger than the ones in experiment (b) and (c). Also, the temperature of the exhaust gas inside the sampling tube increased and decreased but was buffered by the heating and cooling of the material from which the tail pipe was made. Consequently, the temperature peaks were delayed in comparison with the CO_2_ peaks;
- **Experiment (b):** During the transition between experiment (a) and (b), the generator was off, and the pump in the extraction setup was switched on for the entire experiment. During that period, the temperature and the pressure slowly decreased over time following an exponentially decaying function. The pressure only reached a stable value at the end of experiment (b) of about 1100 mbar. When the generator was switched on for the first time (see the blue rectangle in the middle of Figure 11), the CO_2_ concentration was raised up and reached 60,000 ppm. The temperature rose with a delay. The temperature delay was observed in the three experiments. The same behavior as in experiment (a) was observed: temperature and CO_2_ increased while O_2_ decreased by 4%. The relative humidity remained stable. As in the previous experiment, the generator was turned on and off three times, and this resulted in three CO_2_ peaks that are sharper than the ones in the previous experiment;
- **Experiment (c):** The pump was deactivated during the transition between experiment (b) and (c) while the generator was still running. Therefore, the pressure increased and reached the pressure of experiment (a), which was around 1300 mbar. Experiment (c) started by switching on the pump again and increasing the load by coupling three electric heaters of 6 kW in total to the electric generator. From that point onwards, the pressure started to drop down to 1100 mbar, the CO_2_ concentration rose, and the oxygen concentration dropped. Also, the temperature increased while the relative humidity fluctuated around 52%. The load was activated and deactivated three times while the generator remained on. As a consequence, the sudden disconnection of the load increased the angular velocity of the generator. This resulted in three CO_2_ peaks in the blue rectangle to the right in Figure 11. The first CO_2_ peak is lower than the next two peaks because the load was only connected for a short period. When the load was applied for a longer period, the CO_2_ concentration started to increase and reached the saturation point of the sensor. The experiment was stopped before that point was reached.

## 5. Conclusions

It is possible to build a low-cost, multi-purpose data logger around an Arduino Mega 2560 single-board computer. The data logger was used to perform monitoring campaigns for outdoor air quality, human activity in an office, motion of a journey on a bike, and exhaust gas monitoring of a diesel generator. All case studies show a very dynamic pattern in the trends. The explanations given to some of the events observed in these trends indicate that low-cost data loggers are able to generate meaningful information in the different domains. Despite the possibility to acquire meaningful information, the usability of such monitoring devices in scientific research is often questioned. Such doubts often result in an evaluation of the sensors and/or in different kinds of calibration experiments. This contribution has shown that it is not sufficient to evaluate the quality of the sensors and their calibration but that the credibility of the entire system must be evaluated as well. Several aspects of such an evaluation framework can be found in the literature and standards, but an overarching framework from the point of view of the entire system seemed to be missing. Therefore, a credibility assessment process and an evaluation framework consisting of targets and key performance indicators was proposed. The targets were grouped in three levels: (1) the device, (2) the sensors, and (3) the data. 

In almost all of the presented campaigns performed with the same low-cost data logger, unexpected events occurred. Given the regular occurrence of unexpected events, it is hard to believe that this is the only work where unexpected events occurred during the development of low-cost data logger systems. As a result, short-term evaluations are not a good estimation for long-term reliability of low-cost scientific devices. The occurrence of unexpected events, or events that are not supposed to happen, makes the development of a low-cost, multi-purpose data logger harder than expected, and more importantly, it makes it hard to counter the belief that low-cost scientific devices are insufficiently reliable to use them for research purposes. Designing data loggers for research purposes is time-consuming. In addition, there is no clear go/no-go decision point to terminate the evaluation process. This is a limitation for any cyclic process that assesses the credibility of low-cost scientific devices. Another limitation is that not all key performance indicators can easily be quantified through measurements. Some indicators can only be assessed through empirical observation of the behavior of the system during a monitoring campaign.

Unexpected events are only unexpected when they occur for the first time. As the evaluator encounters more of these events, he can build a growing database of scenarios that allows him to put the prototype under stress in a more dedicated way. This allows him to observe how it will react to all these stressors. This modus operandi tends more towards failure mode and effects analysis (FMEA) and test-driven design. When users report unexpected events to the evaluator, the social aspect of believing in technology is not entirely outside the sphere of control of the developers/evaluators anymore. This means that collecting scenarios where previous prototypes failed is an important action in the continuous evaluation of the data logger. 

## Data Availability

All data used in this study are available upon request.
